# Mr. M'Cullock on Blood-Letting in Fever

**Published:** 1811-08

**Authors:** Sam. M'Cullock

**Affiliations:** Member of the Royal Physical Society, Edinburgh. Liverpool


*To the Editors of the Medical and Physical Journal.*

GENTLEMEN,

CONSIDERABLE difference of opinion has originated amongst medical men respecting the propriety of blood-letting, as a remedy in fever. This practice has been considered by most physicians as highly injurious: they assert, that the disease arises solely from certain sedative powers acting upon the brain or nervous system, diminishing its energy, and consequently deranging all the rest of the animal functions; and that depletion would therefore only aggravate the symptoms, by inducing a greater degree of debility.

It is upon this supposition that the celebrated Dr. Cullen has founded his ingenious hypothesis, of the remote and proximate causes of fever. According to him, the remote cause of fever depends upon certain sedative powers acting, as above mentioned, by producing debility, which is considered the proximate cause of fever. That a derangement of the nervous system (having in its consequences general debility, and depraved action) is the cause of fever, I will not deny; but that this debility depends upon the sedative powers of contagion, I cannot conceive. Surely it is more consonant with daily observation to suppose, that contagion acts as a stimulus; and that we are to look for debility, with fever and its phenomena, from the natural effects stimuli produce on the system.

That contagion acts as a stimulus is fully proved, I think, in the instances of patients, who have been subjected to the direct influence of the contagious principle.

In these we find, not as is believed, an immediate debility produced, but a very high degree of excitement; as urgent sickness, increased action of the heart and arteries, with pains of the head more or less violent; after which succeed the cold and hot fits, great debility, with all the concomitant symptoms of fever. Taking it then for granted, that contagion acts as a stimulus upon the system; as we are acquainted with its peculiar powers, we may imagine, from the nature of the symptoms (prior to debility), that it has a powerful effect in determining to the brain, and there producing a turgescence of the blood-vessels; which I think will readily account for the different symptoms that occur in typhus.

---

we have occasionally seen considerable benefit derived in cases of amenorrhœa, that had resisted steel and the usual emmenagogues. Further communications on this subject are earnestly requested.

EDITORS.

typhus. For it is evident that the brain requires a certain fulness, and tension of its vessels, for the distribution of its nervous power; and which is also necessary to the support of its ordinary and constant energy. Now as a deficiency of tension or fulness, for obvious reasons, will induce debility, so I conceive, that a turgescence of the vessels of the brain does not produce increased action; but rather, by their over distension, and by their pressure upon the sentient organ, diminish its mobility, and of course induce general debility.

That a turgescence of the blood-vessels of the brain does take place in this disease, is fully corroborated by the dissections which have been made upon those persons who have fallen victims to it; and in almost every instance it has been found, either in a lesser or greater degree.

Not unfrequently also, an apparent inflammation of the brain has been observed, which may account for that high degree of delirium, which so often takes place in persons labouring under the disease, and which may be considered, in fact, as a modification of phrenitis. Again, in this disease, if the eyes are examined, the blood-vessels will be generally found uncommonly turgid.

If what has been now stated be correct, little doubt can remain, I think, respecting the propriety of blood-letting; and therefore, our first consideration ought to be, the removal of the exciting, or rather, I may say, the depressing cause, and by that means induce reaction; but nothing will effect this so completely as depletion.

I am, Gentlemen, yours, &c.

SAM. M'CULLOCK,
Member of the Royal Physical Society, Edinburgh.

*Liverpool, May* 1811.

